# Identification of a six-gene signature predicting overall survival for hepatocellular carcinoma

**DOI:** 10.1186/s12935-019-0858-2

**Published:** 2019-05-21

**Authors:** Gao-Min Liu, Hua-Dong Zeng, Cai-Yun Zhang, Ji-Wei Xu

**Affiliations:** Department of Hepatobiliary Surgery, Meizhou People’s Hospital, No. 38 Huangtang Road, Meizhou, 514000 China

**Keywords:** Hepatocellular carcinoma, TCGA, GEO, Prognosis, Gene signature

## Abstract

**Background:**

Hepatocellular carcinoma (HCC) remains a major challenge for public health worldwide. Considering the great heterogeneity of HCC, more accurate prognostic models are urgently needed. To identify a robust prognostic gene signature, we conduct this study.

**Materials and methods:**

Level 3 mRNA expression profiles and clinicopathological data were obtained in The Cancer Genome Atlas Liver Hepatocellular Carcinoma (TCGA-LIHC). GSE14520 dataset from the gene expression omnibus (GEO) database was downloaded to further validate the results in TCGA. Differentially expressed mRNAs between HCC and normal tissue were investigated. Univariate Cox regression analysis and lasso Cox regression model were performed to identify and construct the prognostic gene signature. Time-dependent receiver operating characteristic (ROC), Kaplan–Meier curve, multivariate Cox regression analysis, nomogram, and decision curve analysis (DCA) were used to assess the prognostic capacity of the six-gene signature. The prognostic value of the gene signature was further validated in independent GSE14520 cohort. Gene Set Enrichment Analyses (GSEA) was performed to further understand the underlying molecular mechanisms. The performance of the prognostic signature in differentiating between normal liver tissues and HCC were also investigated.

**Results:**

A novel six-gene signature (including CSE1L, CSTB, MTHFR, DAGLA, MMP10, and GYS2) was established for HCC prognosis prediction. The ROC curve showed good performance in survival prediction in both the TCGA HCC cohort and the GSE14520 validation cohort. The six-gene signature could stratify patients into a high- and low-risk group which had significantly different survival. Cox regression analysis showed that the six-gene signature could independently predict OS. Nomogram including the six-gene signature was established and shown some clinical net benefit. Furthermore, GSEA revealed several significantly enriched oncological signatures and various metabolic process, which might help explain the underlying molecular mechanisms. Besides, the prognostic signature showed a strong ability for differentiating HCC from normal tissues.

**Conclusions:**

Our study established a novel six-gene signature and nomogram to predict overall survival of HCC, which may help in clinical decision making for individual treatment.

**Electronic supplementary material:**

The online version of this article (10.1186/s12935-019-0858-2) contains supplementary material, which is available to authorized users.

## Background

Hepatocellular carcinoma (HCC) is the fifth leading cause of malignant cancer and the third most common cause of cancer-related death worldwide [1]. Despite the great improvement in earlier diagnosis and multidisciplinary cancer management, the long-term prognosis remains poor. Thus, an effective prognostic model that identify patients with a high risk of recurrence and metastasis could guide clinical management. Conventional models utilizing clinical tumor-node-metastasis (TNM) staging, vascular invasion, and other parameters help predict HCC prognosis [2]. However, considering the great heterogeneity of HCC, the predictive efficacy of conventional models is still far from satisfying. It’s important to take molecular markers into key account when establishing novel predictive tools.

With the advance of genome-sequencing technologies, accumulating evidence shown that gene signatures at mRNA level had great potential in predicting HCC prognosis. For example, Long et al. established a four-gene-based prognostic model (including gene CENPA, SPP1, MAGEB6, and HOXD9) that accurately predicted overall survival OS using data from The Cancer Genome Atlas-Liver Hepatocellular Carcinoma Dataset (TCGA-LIHC) [3]. Similarly, Zheng et al. identified another four-gene-based signature (including gene SPINK1, TXNRD1, LCAT, and PZP) for predicting the prognosis of HCC using data from the TCGA-LIHC and gene expression omnibus (GEO) database [4]. Deep mining of publicly available genomic data tends to be an efficient method to identify novel robust gene prognostic signatures to guide patients’ prognostic stratification and personalized therapy.

In this study, we conduct univariate and lasso Cox regression analysis to identify novel prognostic biomarkers and established a prognostic six-gene signature using data from TCGA. Multivariate Cox regression analysis confirmed the independent prognostic role of our six-gene signature. Nomogram was established to predict HCC prognosis. Gene set enrichment analysis was performed to help explain the intrinsic mechanisms. In addition, the prognostic value of our six-gene signature was further validated in GSE14520 dataset from GEO database. Besides, the prognostic signature showed a strong ability for differentiating HCC from normal tissues. Collectively, our results suggest the six-gene signature and nomogram might help effectively predict overall survival of HCC patients.

## Materials and methods

### Data collection

Level 3 mRNA expression and clinical data from 374 LIHC and 50 normal control samples were obtained from TCGA-LIHC and cBioportal for Cancer Genomics [5, 6]. Data were downloaded from the publicly available database hence it was not applicable for additional ethical approval.

### Identification of differentially expressed mRNA in HCC

The raw count data were firstly normalized with transcripts per million (TPM) method and underwent a log2 transformation. Then 19654 protein-coding genes were annotated. The differentially expressed mRNA (DEMs) were calculated using the Limma version 3.36.2 R package [7]. DEMs with an absolute log2 fold change (FC) > 1 and an adjusted P value of < 0.05 were considered for subsequent analysis.

### Establishment of the prognostic gene signature

Only patients with a follow-up period longer than 1 month were included for survival analysis. Univariate Cox regression analysis was performed to identify prognostic genes, and genes were considered significant with a cut-off of P < 0.001. Then patients were randomly separated into a training set and testing set. Lasso-penalized Cox regression analysis was conducted to further select prognostic genes for OS in patients with HCC [8]. Then a prognostic gene signature was constructed based on a linear combination of the regression coefficient derived from the lasso Cox regression model coefficients (β) multiplied with its mRNA expression level. The risk score = (β_mRNA1_ * expression level of mRNA1) + (β_mRNA2_ * expression level of mRNA2) + (β_mRNA3_ * expression level of mRNA3) + ⋯ + (β_mRNAn_ * expression level of mRNAn). The optimal cut-off value was investigated by The R package “survival” [7] and “survminer” and two-sided log-rank test. Patients were classified into a high-risk and low-risk cohort according to the threshold. The time-dependent receiver operating characteristic (ROC) curve was drawn to evaluate the predictive value of the prognostic gene signature for overall survival using the R package “survivalROC” [9]. The Kaplan–Meier survival curve combined with a log-rank test was used to compare the survival difference in the high- and low-risk group using the R package “survival”. Then the predictive value of the prognostic gene signature was further investigated in the testing cohort and the whole cohort.

### External validation of the prognostic gene signature and gene expression pattern

GSE14520 dataset from the GEO database was downloaded [10]. The risk score for each included patient was calculated with the same prognostic gene-signature based model. Next, the ROC curve and the Kaplan–Meier curve were used to test the predictive value of the prognostic gene signature. The mRNA expression of the genes in the prognostic gene signature was analyzed in HCC and non-tumor tissue using the Wilcoxon signed-rank test. The two-sided P < 0.05 was considered statistically significant. The protein expression of the genes in the prognostic gene signature was explored in the Human Protein Atlas (http://www.proteinatlas.org) online database.

### Independent prognostic role of the gene signature

To investigate whether the prognostic gene signature could be independent of other clinical parameters [including gender, age, body mass index (BMI), alpha-fetoprotein (AFP), tumor grade, inflammation, vascular tumor invasion, and TNM stage], univariate and multivariate analyses were performed using the Cox regression model method with forwarding stepwise procedure. P < 0.05 were considered as statistically significant.

### Building and validating a predictive nomogram

Nomogram is widely used to predict cancer prognosis [11]. All independent prognostic factors identified by multivariate Cox regression analysis were included to build a nomogram to investigate the probability of 1-, 3-, and 5-OS of HCC. Validation of the nomogram was assessed by discrimination and calibration. The concordance index (C-index) was calculated to assess the discrimination of the nomogram by a bootstrap method with 1000 resamples. The calibration curve of the nomogram was plotted to observe the nomogram prediction probabilities against the observed rates. Subsequently, we compared the nomogram including all with those including only one independent prognostic factor using the time-ROC curve, C-index, and the decision curve analysis (DCA) [12]. DCA was used to calculate the clinical net benefit of each model compared to all or none strategies. The best model is the one with the highest net benefit as calculated.

### Gene Set Enrichment Analyses

To explore the potential molecular mechanisms underlying our constructed prognostic gene signature, GSEA (Gene Set Enrichment Analyses) [13, 14] was performed to find enriched terms predicted to have a correlation with the Kyoto Encyclopedia of Genes and Genomes (KEGG) pathway in C2; in C5, a gene set that contain genes annotated by the same gene ontology (GO) term; and in C6, oncogenic signatures of gene sets often dysregulated in cancer. P < 0.01 and FDR (false discovery rate) q < 0.05 were considered statistically significant.

### Differentiating performance of the prognostic signature

Boxplot and ROC curve was used to explore the difference of the risk score and the differentially diagnostic capability of the risk score between normal liver and HCC, respectively. P < 0.05 was considered statistically significant.

### Statistical analysis

Statistical analyses were performed using R software v3.5.0 (R Foundation for Statistical Computing, Vienna, Austria) and GraphPad Prism v7.00 (GraphPad Software Inc., USA). Qualitative variables were analyzed using the Pearson χ2 test or Fisher’s exact test; quantitative variables were analyzed using a t-test for paired samples or a non-parametric Wilcoxon rank-sum test for unpaired samples as appropriate. Multiple groups of normalized data were analyzed using one-way ANOVA. If not specified above, P < 0.05 was considered statistically significant.

## Results

### DEMs identification

**Fig. 1:**
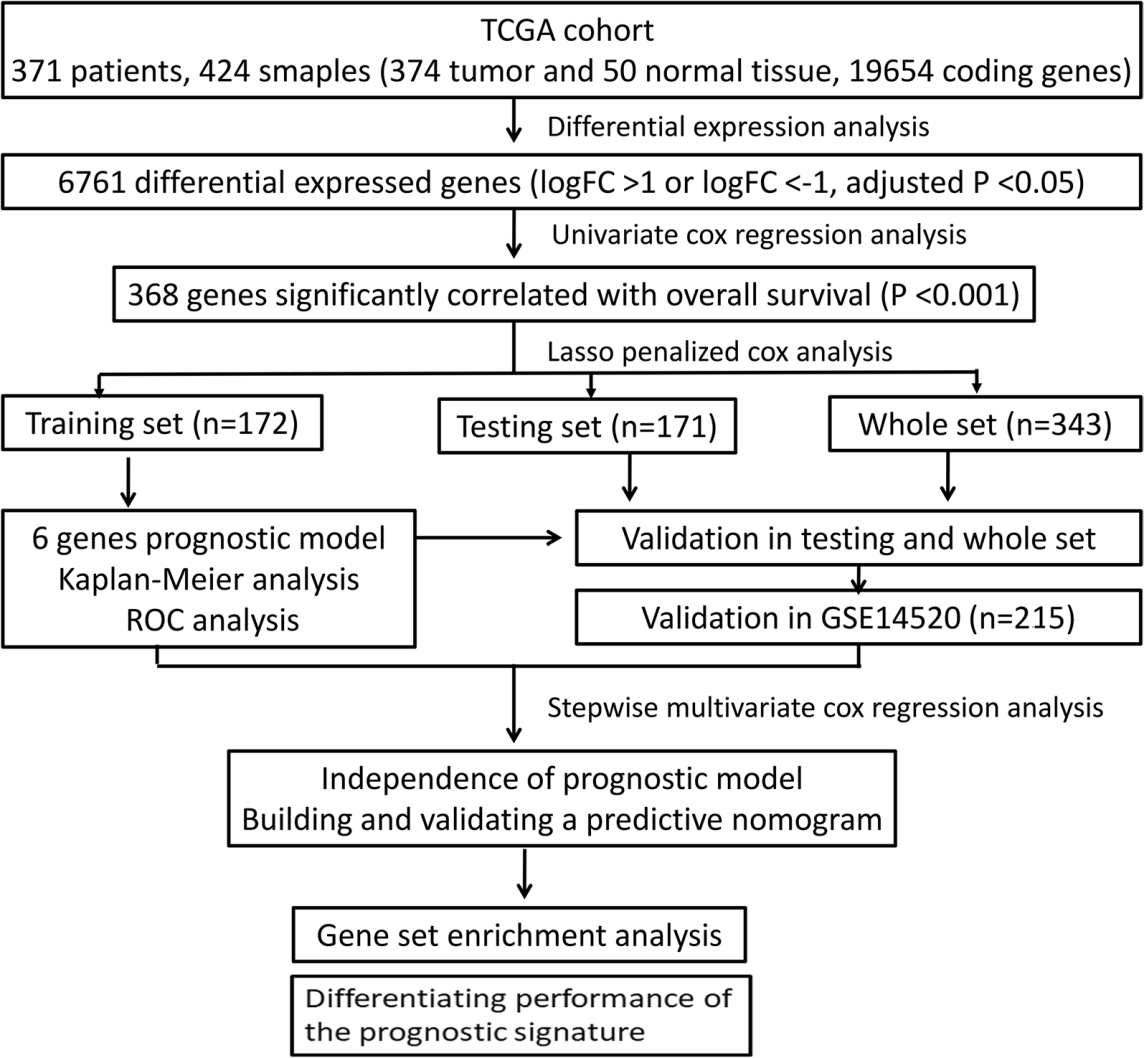
The flow chart showing the scheme of our study on mRNA prognostic signatures for hepatocellular carcinoma

We conducted our study as described in the flow chart (Fig. 1). A total of 6761 genes were identified as differentially expressed at mRNA level in tumor tissues (n = 374) when compared with that of normal tissues (n = 50). The heatmap of the DEMs was shown in Additional file 1: Figure S1. mRNA of 5822 genes were found to be significantly up-regulated, while that of 939 genes were found to significantly down-regulated (Additional file 2: Figure S2).

### Establishment of the six-gene-based prognostic gene signature

**Fig. 2:**
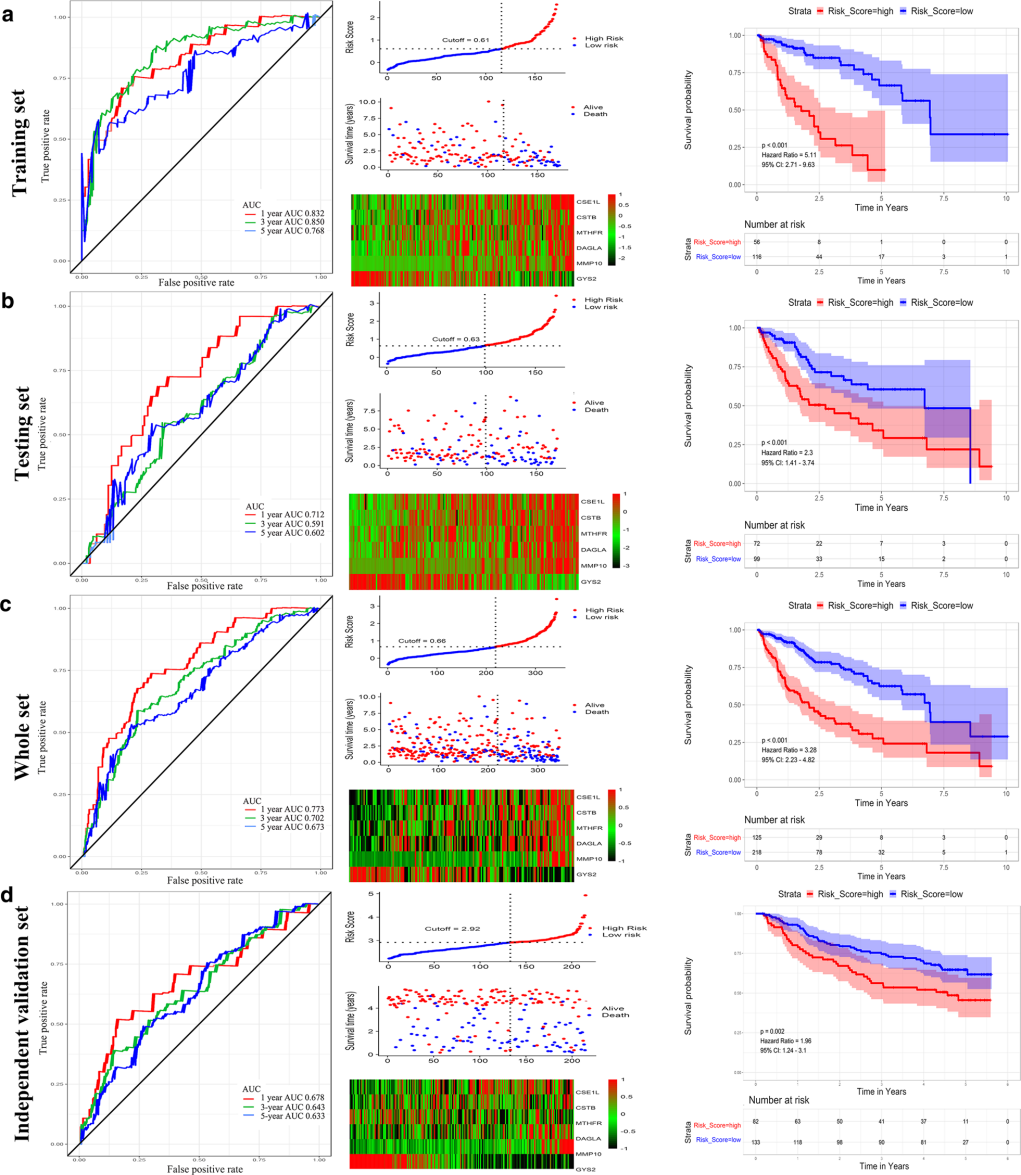
Time-dependent ROC analysis, risk score analysis, and Kaplan–Meier analysis for the six-gene signature in HCC. **a** Time-dependent ROC analysis, risk score, heatmap of mRNA expression, and Kaplan–Meier curve of the six-gene signature in the training set of TCGA cohort. **b** Time-dependent ROC analysis, risk score, heatmap of mRNA expression, and Kaplan–Meier curve of the six-gene signature in the testing set of TCGA cohort. **c** Time-dependent ROC analysis, risk score, heatmap of mRNA expression, and Kaplan–Meier curve of the six-gene signature in the whole included set of TCGA cohort. **d** Time-dependent ROC analysis, risk score, heatmap of mRNA expression, and Kaplan–Meier curve of the six-gene signature in GSE14520 cohort. *HCC* hepatocellular carcinoma, *ROC* receiver operating characteristic, *TCGA* The Cancer Genome Atlas

343 patients with a follow-up period longer than 1 month were included for subsequent survival analysis. Then patients were randomly separated into a training set (n = 172) and testing set (n = 171). The baseline characteristics were summarized in Additional file 3: Table S1. No clinical parameters except adjacent hepatic tissue inflammation type were significantly different in the training set and testing set. Univariate Cox regression model identified 368 genes that significantly associated with OS. Then, lasso-penalized Cox analysis was performed in the training set (n = 171) to further narrow the mRNAs (Additional file 4: Figure S3). Six genes were identified and subsequently used to construct a prognostic gene-signature. The six genes identified were chromosome segregation 1-like (CSE1L), cystatin B (CSTB), methylenetetrahydrofolate reductase (MTHFR), diacylglycerol lipase alpha (DAGLA), matrix metalloproteinase 10 (MMP10), and glycogen synthase 2 (GYS2). The risk score = 0.0606 * Expression_CSE1L_ + 0.0257 * Expression_CSTB_ + 0.1177 * Expression_MTHFR_ + 0.1912 * Expression_DAGLA_ + 0.4324 * Expression_MMP10_ + (− 0.1003) * Expression_GYS2_. We then calculated the six-gene based risk score for each patient and used the Survminer R package to find the optimal cut-off for the risk score. Time-dependent ROC and Kaplan–Meier curve were used to assess the prognostic capacity of the six-gene signature. Similar procedures were performed in the testing set and the whole set. The AUCs (Area under the ROC curve) for 1-year, 3-year, and 5-year OS were 0.832, 0.850, 0.768, and 0.712, 0.591, 0.602, and 0.773, 0.702, 0.673 for training set, testing set, and whole set, respectively. Patients in the high-risk group shown significantly poorer OS than patients in the low-risk group (all P < 0.001) (Fig. 2a–c). Compared with other six signatures [3, 4, 15–18], our signature showed a middle C-index and comparable AUCs for 1-, 3-, 5-year OS prediction (Additional file 5: Figure S4; Additional file 6: Table S2). Collectively, our results indicated a good performance of the six-gene signature for survival prediction.

### External validation of the prognostic gene signature

To validate the predictive value of the six-gene signature, we calculated risk score with the same formula for patients in GSE14520. Consistent with the results in the TCGA cohort, patients in the high-risk group shown significantly poorer OS than patients in the low-risk group (P = 0.002). The AUCs for 1-year, 3-year, and 5-year OS were 0.678, 0.643, and 0.633, respectively (Fig. 2d). Taking together, the six-gene signature was capable of predicting OS in HCC.

### External validation of the genetic alteration and expression of the six gene

**Fig. 3:**
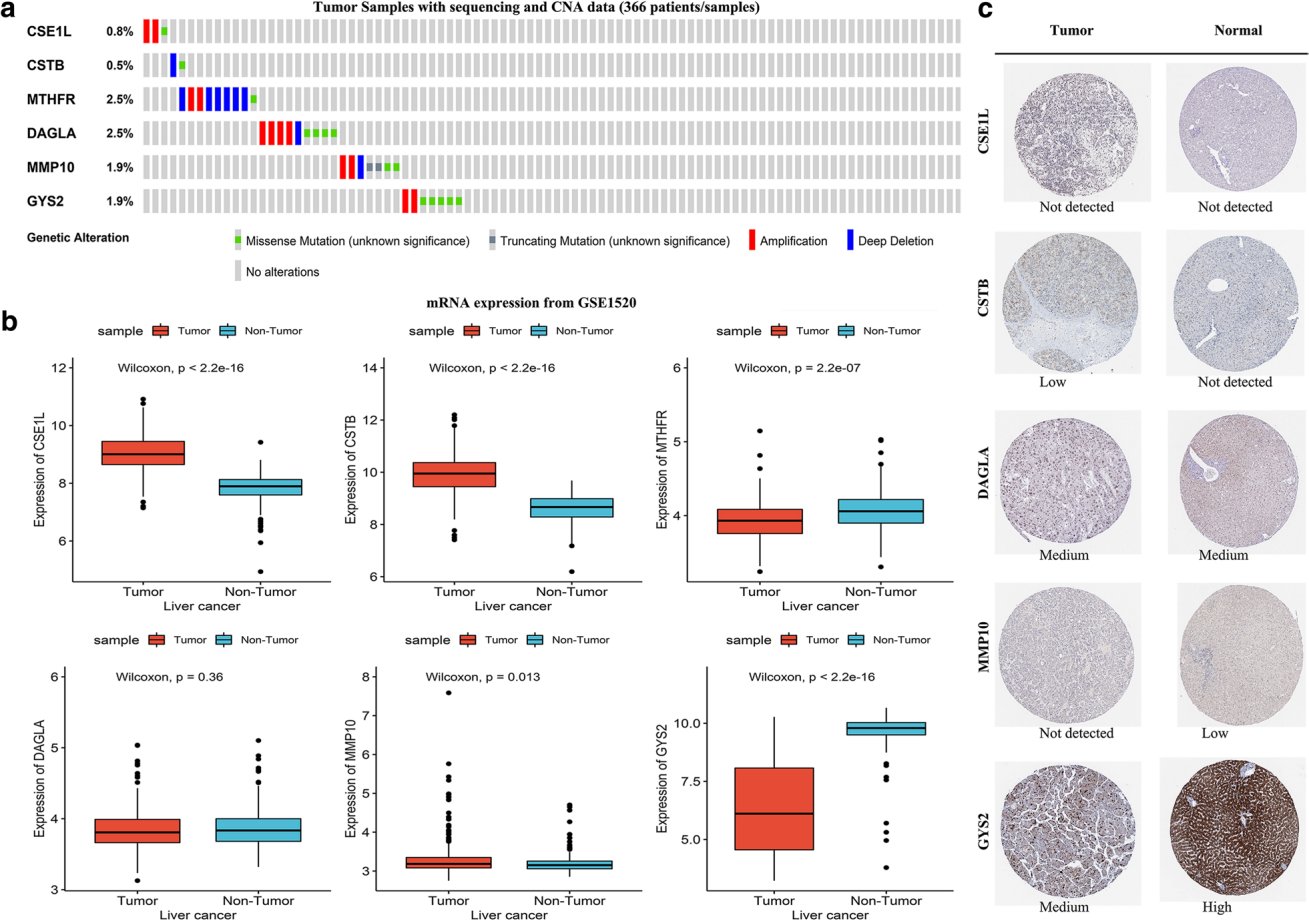
The expression and genetic alterations of the six prognostic genes in HCC. **a** The expression alteration profiles of the six genes in the TCGA liver cancer RNA-seq (n = 366) dataset. **b** The expression profiles of the six genes in the GSE14520 cohort. **c** The representative protein expression of the six genes in HCC and normal liver tissue. Data were from the Human Protein Atlas (http://www.proteinatlas.org) online database. *HCC* hepatocellular carcinoma

Among the 366 patients included in cBioportal for Cancer Genomics database, 36 (10%) shown genetic alterations in the six genes. Missense mutation was the most common genetic alteration (Fig. 3a). Consistent with the results in the TCGA cohort, the mRNA expression of CSE1L, CSTB, MMP10 was significantly up-regulated in HCC while GYS2 was significantly down-regulated when compared with non-tumor tissues. Yet the upregulation of MHTFR and DAGLA was not found in GSE14520 cohort (Fig. 3b). We further explored the protein expression of the six genes in the Human Protein Profiles and shown the characteristic pictures of them in Fig. 3c. However, we did not find MTHFR protein expression in the database.

### Independent prognostic role of the gene signature

**Fig. 4:**
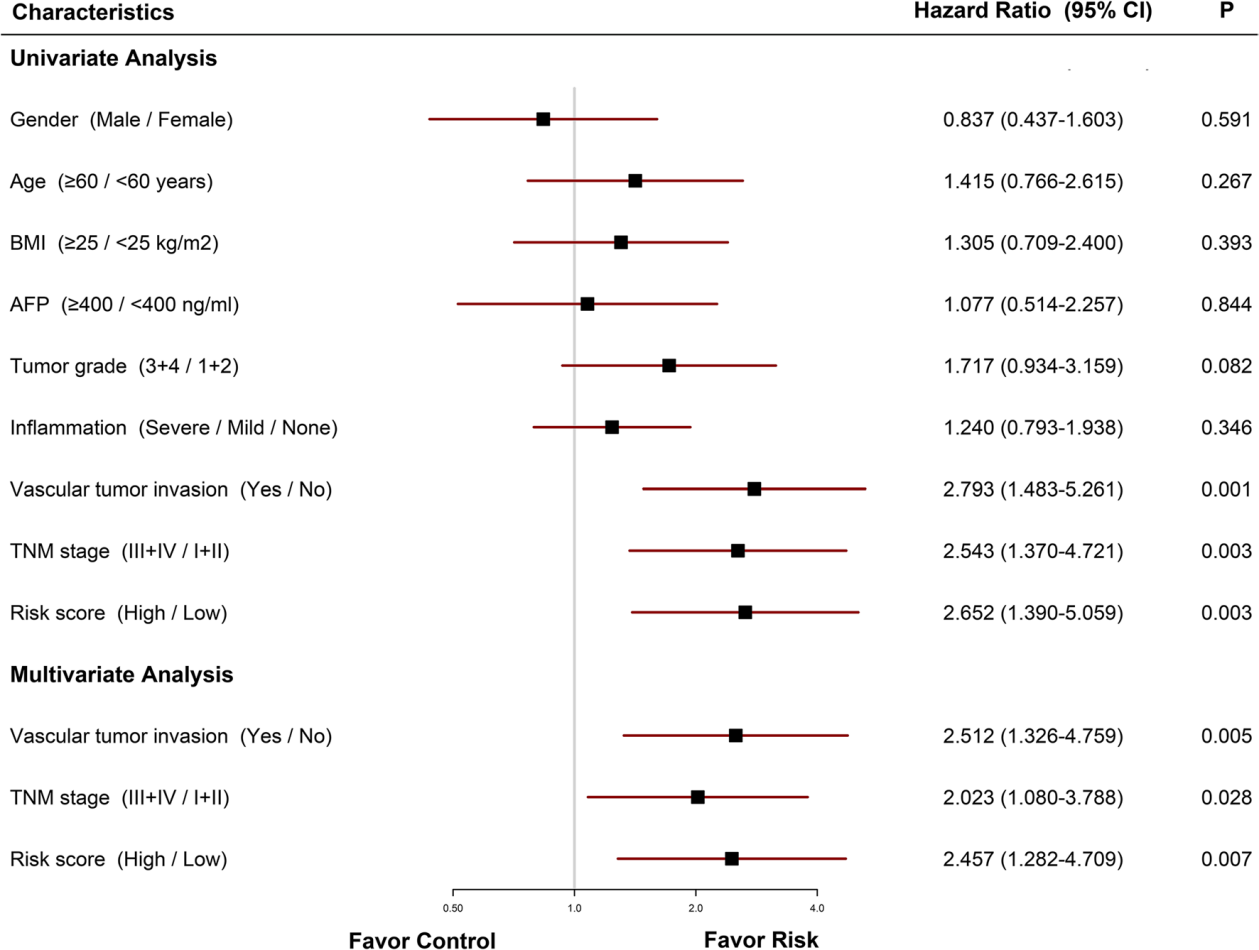
Forrest plot of the univariate and multivariate Cox regression analysis in HCC. *HCC* hepatocellular carcinoma

165 patients with complete information including gender, age, BMI, AFP, tumor grade, inflammation, vascular tumor invasion, and TNM stage were included for further analysis. Univariate and multivariate Cox regression analysis indicated that vascular tumor invasion, TNM stage, and risk score calculated from the six-gene signature were independent prognostic factors for OS (Fig. 4).

### Building and validating a predictive nomogram

**Fig. 5:**
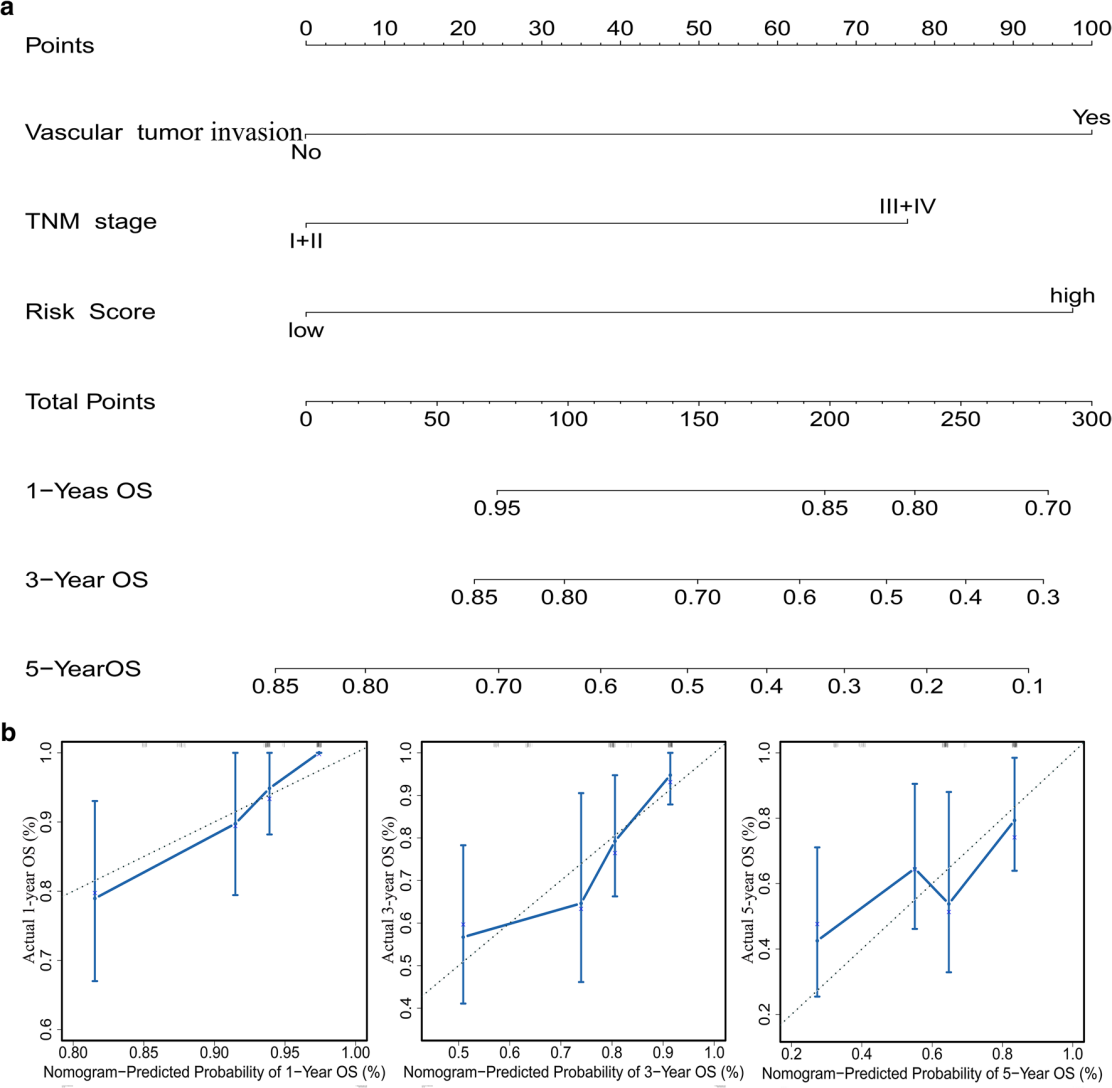
Nomogram predicting overall survival for HCC patients. **a** For each patient, three lines are drawn upward to determine the points received from the three predictors in the nomogram. The sum of these points is located on the ‘Total Points’ axis. Then a line is drawn downward to determine the possibility of 1-, 3-, and 5-year overall survival of HCC. **b** The calibration plot for internal validation of the nomogram. The Y-axis represents actual survival, and the X-axis represents nomogram-predicted survival. *HCC* hepatocellular carcinoma

**Fig. 6:**
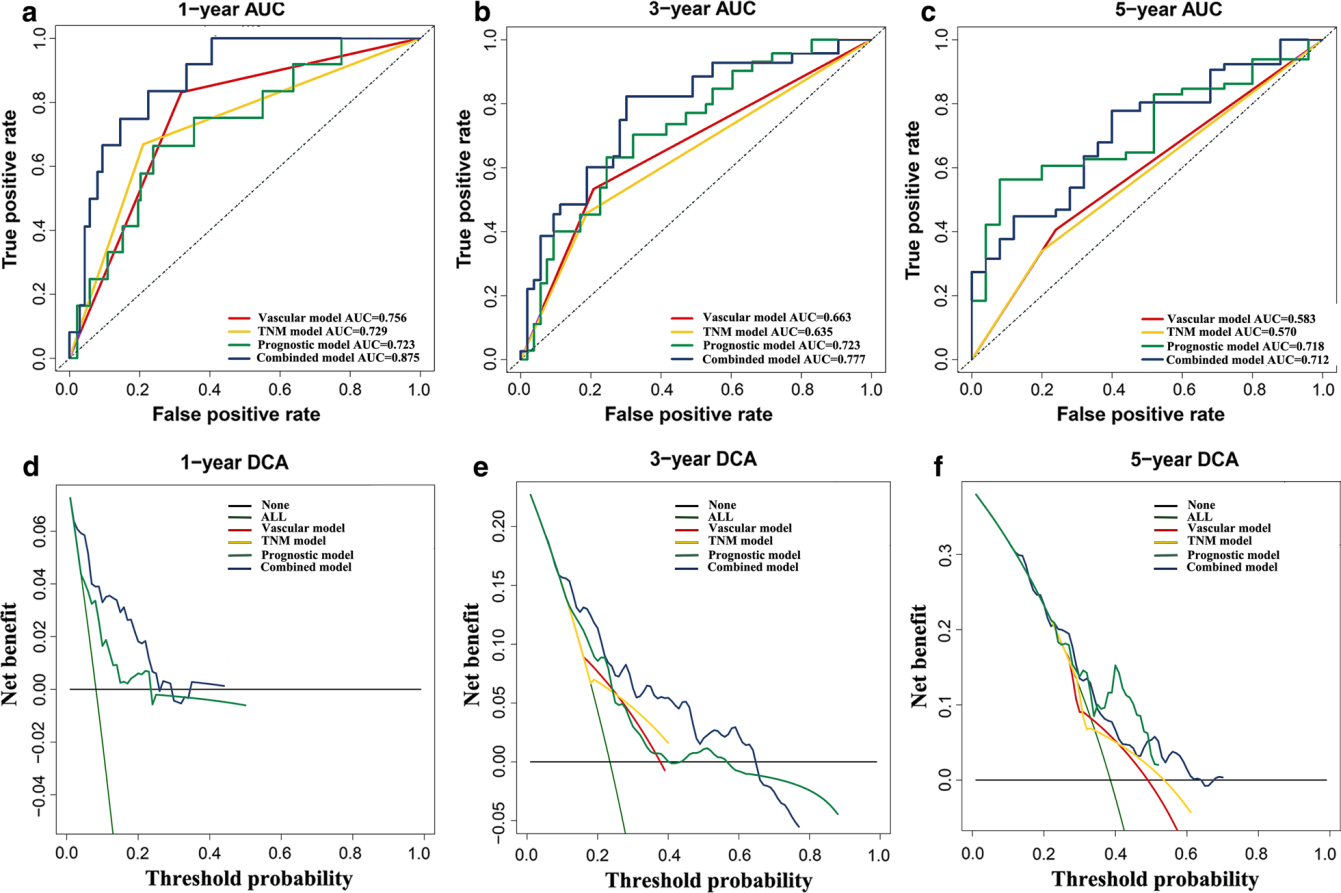
The time-dependent ROC and DCA curves of the nomogram. **a**–**c** The time-dependent ROC curves of the nomograms compared for 1-, 3-, and 5-year overall survival in HCC, respectively. **d**–**f** The DCA curves of the nomograms compared for 1-, 3-, and 5-year overall survival in HCC, respectively. The none plot represented the assumption that no patients have 1-, 3- or 5-year survival; while all plot represented the assumption that all patients have 1-, 3- or 5-year survival at a specific threshold probability. The x-axis represented the threshold probabilities, and the y-axis measured the net benefit. In **d**, the DCA curves of vascular and TNM model were not shown as the calculated net benefit were all smaller than calculated with the none assumption. *ROC* receiver operating characteristic, *DCA* decision curve analysis, *HCC* hepatocellular carcinoma

We then built a nomogram to predict 1-year, 3-year, and 5-year OS in the 165 HCC patients using three independent prognostic factors including vascular tumor invasion, TNM stage, and risk score. Calibration plots showed that the nomogram (combined model) might under-estimate or over-estimate the mortality (Fig. 5). The C-index for vascular tumor invasion, TNM stage, risk score, and the combined model was 0.66 (95% confidence interval [CI] 0.58–0.74), 0.61(95% CI 0.54–0.61), 0.72 (95% CI 0.62–0.82), and 0.77 (95% CI 0.67–0.86), respectively. The AUCs of the nomogram were 0.87 (95% confidence interval [CI] 0.80–0.95), 0.78 (95% CI 0.66–0.88), and 0.71 (95% CI 0.58–0.85) for 1-year, 3-year, and 5-year OS, respectively (Table 1). Compared with nomogram including only the vascular, TNM, or prognostic gene signature, the combined model shown the largest AUC for 1-year and 3-year OS but not for 5-year OS (Table 1, Fig. 6a–c). DCA demonstrated that the combined model showed the best net benefit for 1-year and 3-year OS but not for 5-year OS as well (Fig. 6d–f). Taking together, these results indicated that compared with nomograms built with a single prognostic factor, the nomogram built with the combined model might be the best nomogram for predicting short-term survival (1-year and 3-year) but not for long-term survival (such as 5-year) for patients with HCC, which might help clinical management.

**Table 1:** Comparison of the nomogram with vascular, TNM stage, prognostic model, and the combined model

Models	1-year AUC (95% CI)	P-value	3-year AUC (95% CI)	P-value	5-year ACU (95% CI)	P-value
Vascular model	0.76 (0.64–0.87)	–	0.66 (0.56–0.77)	–	0.58 (0.47–0.70)	–
TNM model	0.73 (0.59–0.87)	–	0.63 (0.53–0.74)	–	0.57 (0.46–0.68)	–
Prognostic model	0.73 (0.58–0.87)	–	0.72 (0.61–0.84)	–	0.72 (0.57–0.86)	–
Nomogram (combined) model	0.87 (0.80–0.95)	–	0.78 (0.66–0.88)	–	0.71 (0.58–0.85)	–
Nomogram vs. vascular model		0.05		0.027		0.01
Nomogram vs. TNM model		0.01		0.01		0.03
Nomogram vs. prognostic model		0.01		0.29		0.93

*AUC* area under curve, *CI* confidence interval, *TNM* tumor-node-metastasis

### Gene Set Enrichment Analyses

To explore the underlying molecular mechanisms of the signature, we conduct GSEA comparing the high-risk group with the low-risk group in 343 TCGA patients of the whole set. In the high-risk group, 4 oncological signatures including Early serum response (CSR), E2F Transcription Factor 1 (E2F1), Rb-P107, and granule cell neuron precursors (GCNP) were enriched; however, no KEGG or GO terms were significantly enriched. In the low-risk group, the enriched KEGG pathways and GO terms were mainly focused on various metabolism process (including fatty acid, retinol, tyrosine, butanoate and so on). However, no oncological signatures were significantly enriched (Additional file 7: Table S3).

### Differentiating performance of the prognostic signature

**Fig. 7:**
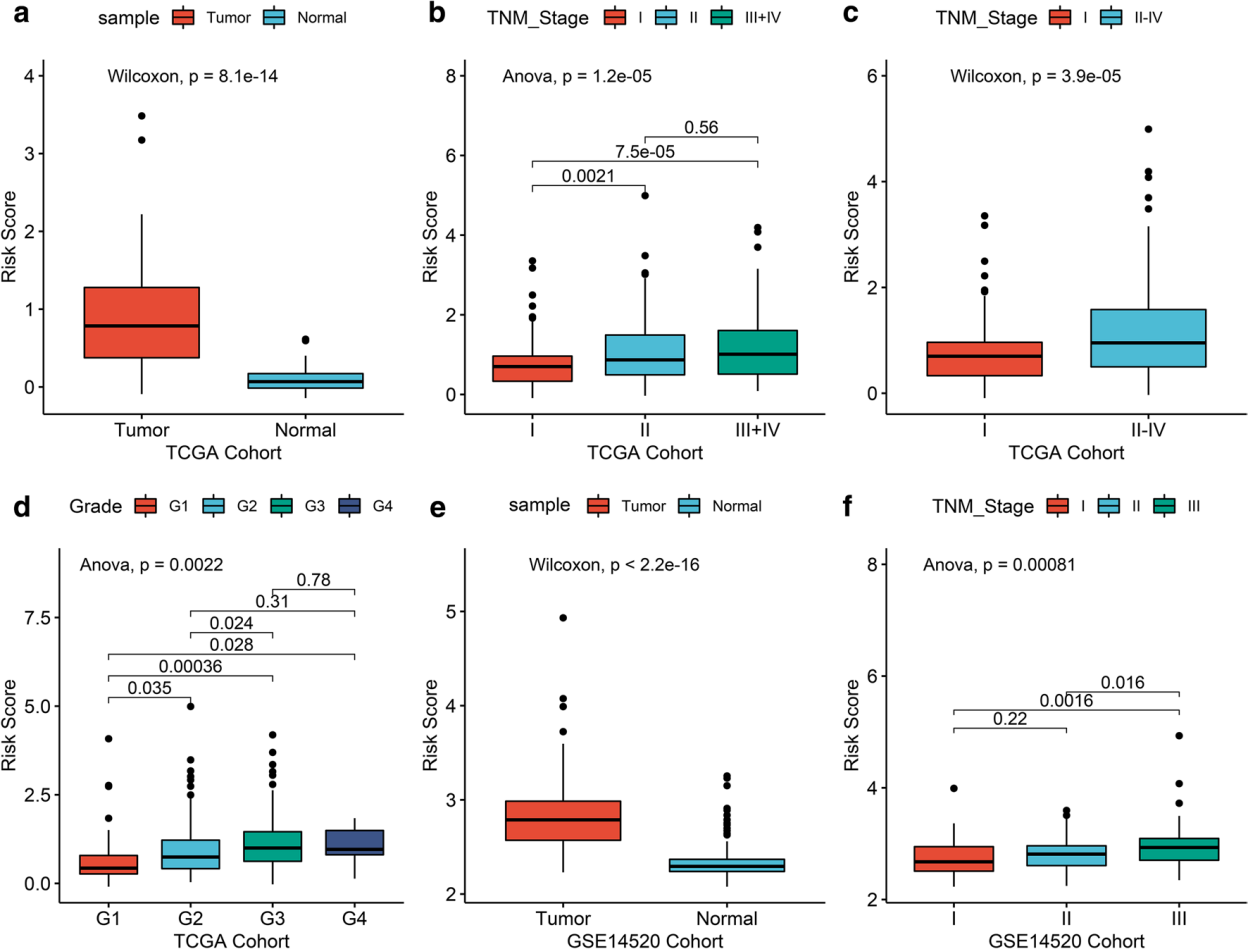
The different risk score in normal tissue and HCC. The risk score was group by **a**, **e** sample type, **b**, **c**, **f** TNM stage, and **d** tumor grade. **a**–**d** Were from the TCGA cohort, while **e**, **f** were from GSE14520 cohort. *HCC* hepatocellular carcinoma

**Fig. 8:**
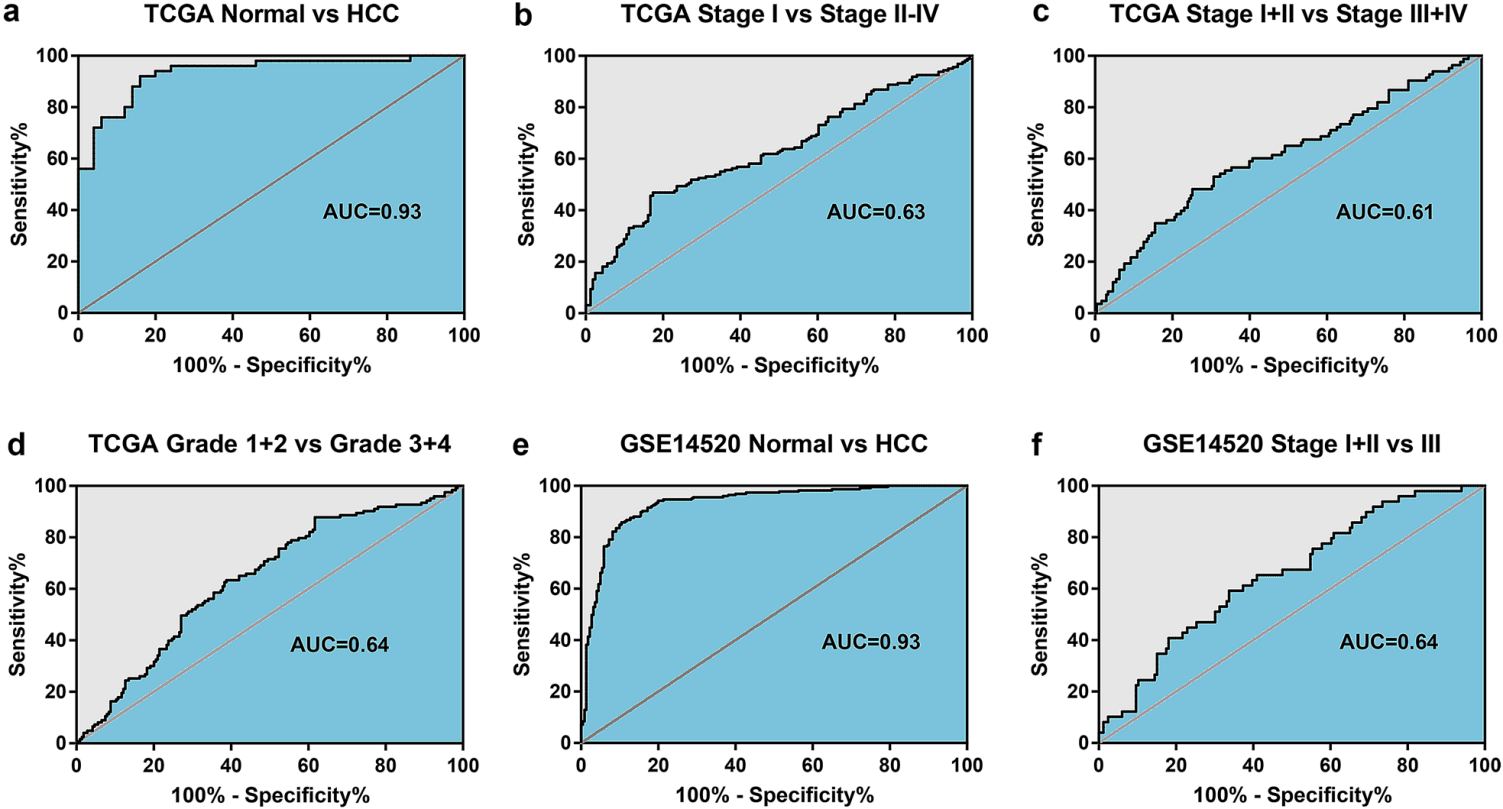
The ROC curve of the risk score in normal tissue and HCC. The ROC curve of the risk score showing its capacity in differentiating between normal and HCC (**a**, **e**), between HCC of different TNM stage (**b**, **c**, **f**), and between HCC of different tumor grade (**d**). *ROC* receiver operating characteristic, *HCC* hepatocellular carcinoma, *TNM* tumor-node-metastasis

The risk score was then compared between normal liver and HCC to explore the differentially diagnostic capability of the prognostic signature. The risk score was found to be significantly higher in HCC when compared with normal control. The risk score was also found to be significantly higher in patients with advanced TNM stage and tumor grade (Fig. 7). The AUC of the risk score was 0.93 in both cohorts, indicating a strong diagnostic ability for HCC. Furthermore, the subgroup analysis of different stages and grades also showed modest diagnostic capability (Fig. 8). Taking together, these results also suggest great potential of the signature in the differential diagnosis of HCC.

## Discussion

HCC remains a major challenge for public health worldwide. Conventional parameters such as TNM staging, vascular invasion, and AFP help predict HCC prognosis in some degree. However, considering the great heterogeneity of HCC, identification of novel prognostic biomarkers and establishment of more accurate prognostic models are urgently needed. And the combination of the prognostic gene signature with conventional clinical parameters may have better predictive efficacy than a single biomarker. Recently, gene-signatures based on aberrant mRNA have gained much attention and shown great potential in prognosis prediction of cancer [3, 16, 17, 19].

In this study, we established a novel six-gene signature (including CSE1L, CSTB, MTHFR, DAGLA, MMP10, and GYS2) for HCC prognosis prediction. While CSE1L, CSTB, MTHFR, DAGLA, and MMP10 were found to be negative prognostic genes, GYS2 was found to do the opposite. The prognosis predictive performance of the signature was good not only in the TCGA HCC cohort but also in the GSE14520 cohort, and comparable with six previously reported models. The six-gene risk was an independent prognostic factor of HCC and patients in the high-risk group shown significantly poorer survival than patients in the low-risk group. ROC and DCA demonstrated that the nomogram combining the six-gene signature and conventional clinical prognostic factors performed the best in predicting short-term survival (1-year and 3-year) but not in long-term survival (such as 5-year) for patients with HCC. All these results indicated that the risk model developed from the six genes could be a useful indicator for HCC survival. Furthermore, GSEA revealed several significantly enriched oncological signatures and various metabolic process, which might help explain the underlying molecular mechanisms of the signature. And we found the risk score shown a strong ability in differentiating HCC from normal tissues, suggesting a great potential of utilizing the signature in HCC differential diagnosis.

CSE1L, also named as CAS (cellular apoptosis susceptibility protein), has been reported as an oncogene in several cancers [20–22]. CSE1L is a multifunctional gene that participates in apoptosis, chromosome assembly, nucleocytoplasmic transport, microvesicle formation, chemo-resistance, and cancer progression [20, 23, 24]. However, the role and mechanism of aberrant CSE1L in HCC remains poorly defined. CSTB is a reversible endogenous inhibitor of lysosomal cysteine proteinases [25]. Mutations of CSTB leads to progressive myoclonus epilepsy (EPM1), which is an inherited and lethal autosomal disease [26]. Dysregulated expression of CSTB has been implicated to be a useful biomarker in various cancers such as ovarian cancer [27], esophageal cancer [28] and breast cancer [29]. Especially, CSTB was found to be overexpressed in most HCCs and was elevated in the serum of most HCC patients [30]. DAGL (Diacylglycerol lipase) hydrolyzes diacylglycerol to 2-arachidonoylglycerol (2-AG) and free fatty acid (FFA) [31]. Disruption of DAGL activity influenced the development of the central nervous system [32]. Recently, Okubo et al. reported that DAGLA promoted tumorigenesis in oral squamous cell carcinomas by regulating cell-cycle [33]. Roy et al. indicated that DAGLA participated in ovarian progression caused by loss of the endosulfatase HSulf-1 [34]. Nevertheless, the role of DAGLA in HCC remains unclear. MTHFR catalyzes the 5,10-methylenetetrahydrofolate to 5-methyltetrahydrofolate, a co-substrate for homocysteine re-methylation to methionine. Methionine is the forebody of *S*-adenosylmethionine (SAM), and SAM is the direct methyl donor for the DNA methylation [35]. Abnormal MTHFR activity leads to abnormal gene methylation, gene instability and finally cancer [36]. Accumulating studies demonstrate that MTHFR polymorphism affects the susceptibility of various cancer, especially HCC [37–41]. Matrix metalloproteinases (MMPs) are widely accepted as critical modulators for tumor microenvironment [42]. MMP10 promoted HCC by involving in tumor angiogenesis, growth, and dissemination [43]. Decreased glycogen concentration negatively correlated with tumor growth [44]. GYS, the rate-limiting enzyme of glycogen synthesis, consists of two isoforms including GYS1 and GYS2. Loss of GYS2 caused glycogen storage disease type 0 [45]. A very recent study revealed that decreased expression of GYS2 reduced glycogen and indicated unfavorable clinical outcomes of HCC. Mechanically, GYS2 suppressed tumor growth in HBV-related HCC via a negative feedback loop with p53 [46].

To our knowledge, the six-gene signature related prognostic model and nomogram have not been reported previously and could be a useful prognostic and diagnostic classification tool of HCC. The risk score was based on mRNA expression but not somatic mutations or methylation status of only six prognostic genes. It could be more routine and cost-effective in practice as it decreased the necessity of whole-genome sequencing for all patients. Nomogram combining our signature with conventional clinical parameters like TNM stage shown significantly improved performance, especially in predicting short-term survival (1-year or 3-year), indicating a more accurate reflection of the great heterogeneity of HCC. However, several limitations of our study should be taken into consideration. Firstly, our study was mainly based on data from TCGA in which most patients were White or Asian. Extending our findings to other ethnic patients should be with great caution. Secondly, external validation of the six-gene signature and prognostic nomogram in more independent cohorts is necessary. Thirdly, the expression and the prognostic role of the six genes at protein level warrant further investigation. Forth, calibration plots showed that the nomogram (combined model) might under-estimate or over-estimate the mortality, efforts should be made to further improve the prediction performance. Fifth, all mechanical analysis in our study was descriptive, further functional experiments are needed to clarify the underlying mechanism of the six genes. Sixth, except its excellent performance in differentiating HCC from normal liver, the performance of our signature in differentiating between the normal liver, liver adenomas, focal nodular hyperplasia, and hepatocellular carcinomas remains to be further elucidated.

## Conclusions

Our study established a novel six-gene signature and nomogram to predict overall survival of HCC, which may help in clinical decision making for individual treatment.

## Additional files

**Additional file 1: Figure S1.** The heatmap of the differentially expressed mRNA in HCC when compared with normal tissue.

**Additional file 2: Figure S2.** Volcano plot shown the expression change in HCC when compared with normal tissue. An absolute log2 fold change (FC) > 1 and an adjusted P value of < 0.05 cutoff was used to defined differentially expressed mRNAs. The red represented significantly up-regulated mRNAs. The blue represented significantly down-regulated mRNAs. The black represented not differentially expressed mRNAs.

**Additional file 3: Table S1.** Clinical features of HCC patients in training set, testing set, and overall set.

**Additional file 4: Figure S3.** LASSO profiles of the 368 prognostic genes in HCC. (A) LASSO coefficient profiles of the 368 prognostic genes in HCC. (B) Lasso deviance profiles of the 368 prognostic genes in HCC.

**Additional file 5: Figure S4.** Comparison of our signature with six previous models using time-dependent ROC analyses.

**Additional file 6: Table S2.** Comparison of our signature with six other signatures reported previously.

**Additional file 7: Table S3.** Gene set enrichment analyses between the high- and low-risk group in 343 TCGA HCC.

## Data Availability

Data sharing not applicable to this article as no datasets were generated or analyzed during the current study.

## Abbreviations

2-AG: 2-arachidonoylglycerol
AFP: alpha-fetoprotein
CSE1L: chromosome segregation 1-like
CAS: cellular apoptosis susceptibility protein
CSTB: cystatin B
C-index: concordance index
BMI: body mass index
DAGL: diacylglycerol lipase
DAGLA: diacylglycerol lipase alpha
DEMs: differentially expressed mRNA
DCA: decision curve analysis
E2F1: E2F Transcription Factor 1
EPM1: Unverricht–Lundborg disease
CSR: early serum response
FDR: false discovery rate
FFA: free fatty acid
FC: fold change
GYS: glycogen synthase
GO: gene ontology
GEO: gene expression omnibus
GSEA: gene set enrichment analyses
GCNP: granule cell neuron precursors
HCC: hepatocellular carcinoma
KEGG: Kyoto Encyclopedia of Genes and Genomes
MMP: matrix metalloproteinase
MTHFR: methylenetetrahydrofolate reductase
OS: overall survival
ROC: receiver operating characteristic
SAM: *S*-adenosylmethionine
TPM: transcripts per million
TNM: tumor-node-metastasis
TCGA-LIHC: The Cancer Genome Atlas-Liver Hepatocellular Carcinoma Dataset
