# Supplementary figures and images for: Identification of a six-gene signature predicting overall survival for hepatocellular carcinoma
Source: Cancer Cell Int. 2019 May 21;19:138. doi: 10.1186/s12935-019-0858-2 (PMC6528264; doi:10.1186/s12935-019-0858-2)

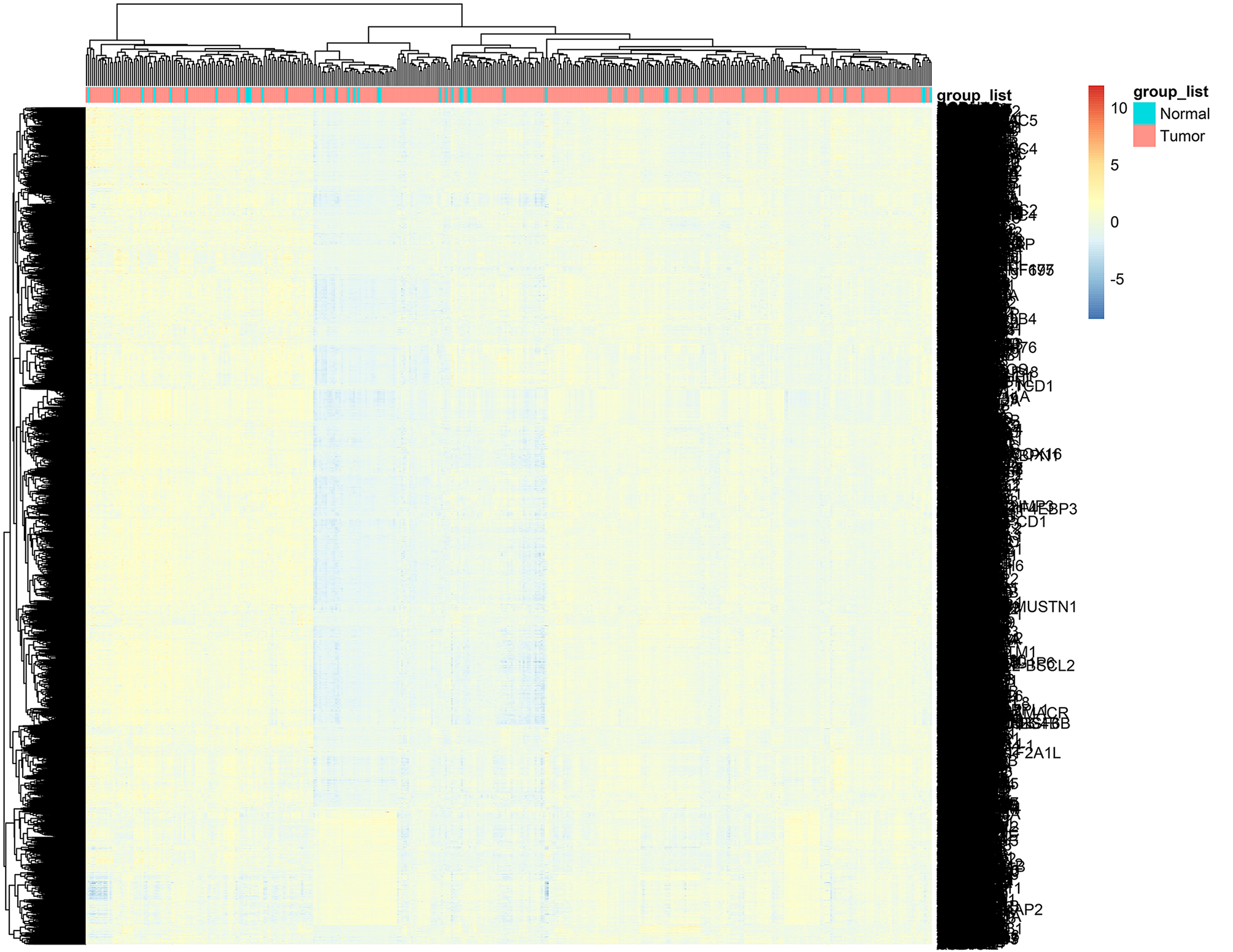

Supplement: Supplementary file 1 — Additional file 1: Figure S1. The heatmap of the differentially expressed mRNA in HCC when compared with normal tissue. [file 12935_2019_858_MOESM1_ESM.tif]

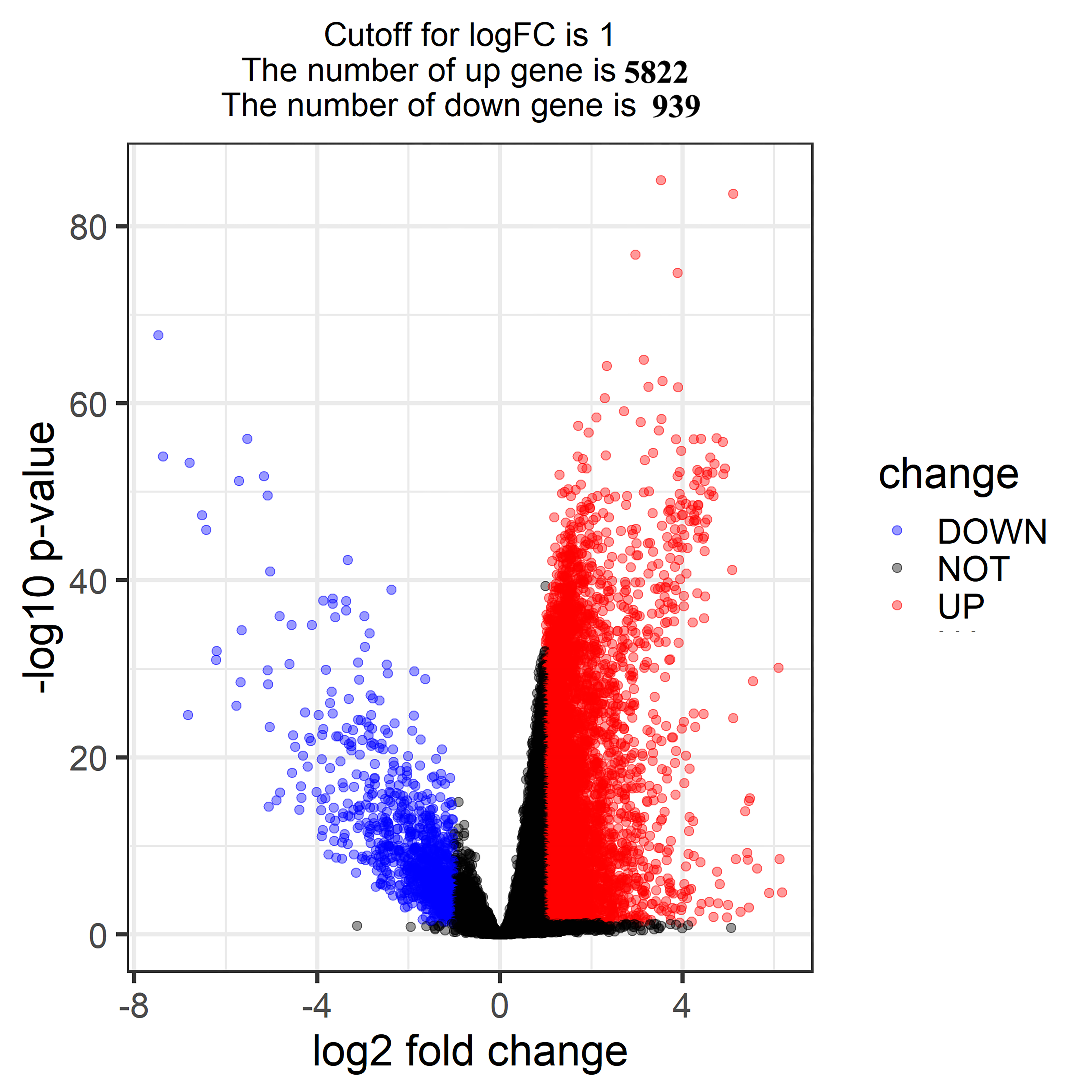

Supplement: Supplementary file 2 — Additional file 2: Figure S2. Volcano plot shown the expression change in HCC when compared with normal tissue. An absolute log2 fold change (FC) > 1 and an adjusted P value of < 0.05 cutoff was used to defined differentially expressed mRNAs. The red represented significantly up-regulated mRNAs. The blue represented significantly down-regulated mRNAs. The black represented not differentially expressed mRNAs. [file 12935_2019_858_MOESM2_ESM.tif]

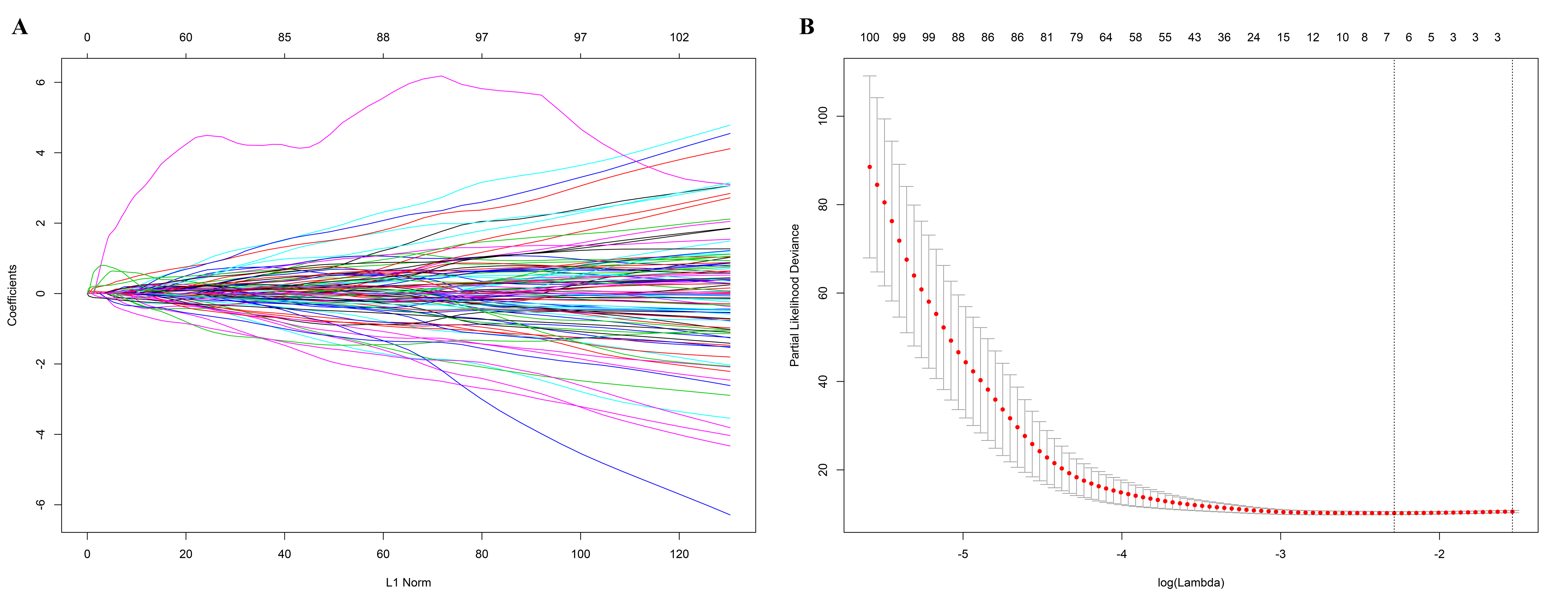

Supplement: Supplementary file 4 — Additional file 4: Figure S3. LASSO profiles of the 368 prognostic genes in HCC. (A) LASSO coefficient profiles of the 368 prognostic genes in HCC. (B) Lasso deviance profiles of the 368 prognostic genes in HCC. [file 12935_2019_858_MOESM4_ESM.tif]

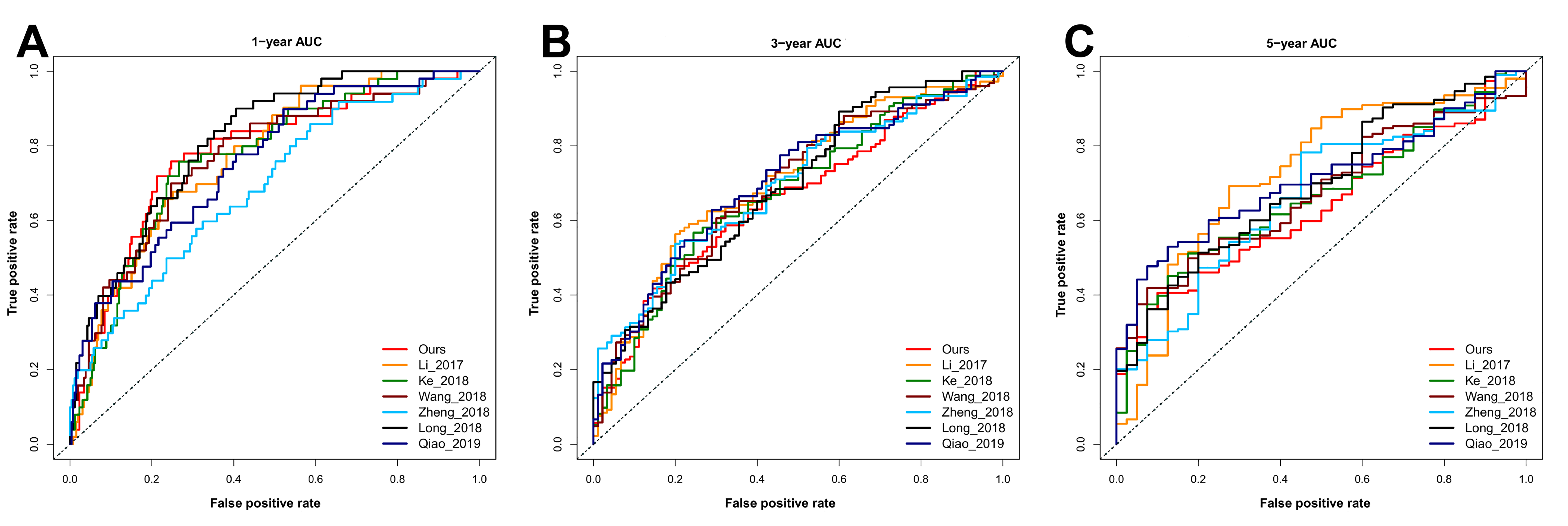

Supplement: Supplementary file 5 — Additional file 5: Figure S4. Comparison of our signature with six previous models using time-dependent ROC analyses. [file 12935_2019_858_MOESM5_ESM.tif]
